# New opportunities at the wild frontier

**DOI:** 10.7554/eLife.06956

**Published:** 2015-03-25

**Authors:** Jane Alfred, Ian T Baldwin

**Affiliations:** Cambridge, United Kingdom; Max Planck Institute for Chemical Ecology, Jena, Germany

**Keywords:** the natural history of model organisms, natural history, model organisms, *Arabidopsis*, *C. elegans*, *E. coli*, *S. cerevisiae*, zebrafish, maize, *Ciona intestinalis*

## Abstract

A better understanding of the natural history of model organisms will increase their value as model systems and also keep them at the forefront of research.

**DOI:** http://dx.doi.org/10.7554/eLife.06956.001

## Introduction

Many of the fundamental principles of biology were discovered by developing a model organism to investigate a biological question. The principles of heredity, the genetic code, transcription, translation and DNA replication were a few of the landmark discoveries made in *Drosophila melanogaster*, the rock-star model insect, and *Escherichia coli*, the model bacterium. But how representative are these models of the ‘rest of life’, as is often assumed? And how do the laboratory lives of model organisms compare with those of their relatives in the wild and those of other closely related species? Surprisingly, we know very little about the real lives of many model organisms, but it is clear that some of them are not very representative of their species.

To explore these questions further, *eLife* has invited a number of researchers to write about the natural histories of some of the best-established model organisms used in biological research, among them the zebrafish *Danio rerio*; the plants, maize and *Arabidopsis thaliana*; the nematode, *Caenorhabitis elegans*; and the microorganisms, *Escherichia* *coli* and *Saccharomyces cerevisiae*. These articles, together with future additions to this series, consider how a better understanding of the natural history of model organisms can extend and enhance their value as model organisms and also keep them at the forefront of cutting edge biological research (http://elifesciences.org/natural-history-of-model-organisms).

By providing a natural history context to these much loved, but decontextualized, research organisms, we hope that the articles in this series will help to heal the unhappy division of biology departments along cellular-molecular-developmental and ecological-evolutionary lines. This split, which is reflected in many graduate training programmes, is largely responsible for the dearth of natural history information about these model organisms. We also hope that these articles will prepare a more unified family of biologists to anticipate the research possibilities of a not-too-distant future, when the distinctions between model and non-model organisms become blurred.

## Supermodels are not always representative

Just as the supermodels of the fashion world do not represent the average *Homo sapiens*, some ‘supermodel organisms’ do not represent their nearest relatives or even their own species. Moreover, some of these supermodel organisms have never lived in the wild. The crop plant maize is one such example, and the conversion of the wild grass teosinte into maize some 7–10,000 years ago is perhaps the first example of biotechnological innovation by humans. The domestication of the yeast *S. cerevisiae* for brewing and baking happened around the same period. House mice and the nematode, *C. elegans*, also share a long evolutionary history with humans, becoming human commensals when our houses and orchards became their habitats, but they do not owe their existence to humans. The relationship of other model organisms with humans is more recent. For example, humans distribute the marine model organism *Ciona intestinalis*—an organism that is of increasing interest to evolutionary biologists because it occupies a key branch point in the evolution of chordates—around the world's oceans via ships.

In many cases, model organisms differ from their closest non-model relatives by possessing particular traits that have facilitated their domestication and adaptation to the laboratory environment, and over time this process has accentuated the differences between model organisms and their wild relatives. However, some model organisms, such as *C. intestinalis*, have not (as yet) been adapted to breed in the laboratory. While this raises certain challenges (data generated from locally collected specimens, for example, might differ significantly from that stored in community databases), it also offers opportunities to study important evolutionary processes, such as adaptation and variation.

**Figure 1.**
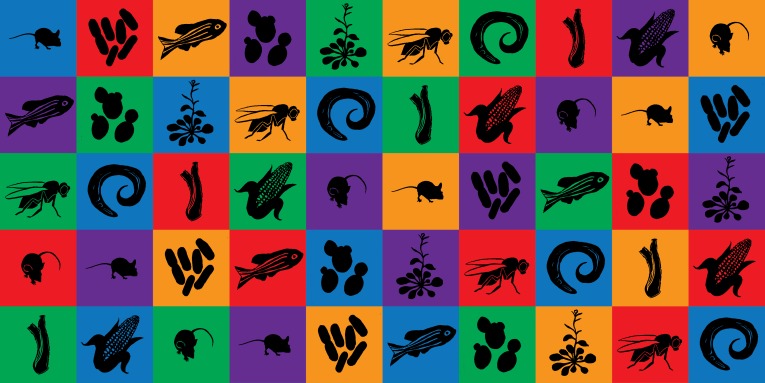
Scientists know a great deal about model organisms as diverse as *Arabidopsis thaliana* and the zebrafish, but there is still a lot to learn about their life and biology in the wild, including the way that their behaviours are shaped by habitat, their predator-prey relationships, their ability to adapt to different environmental conditions, and the genetic basis for this adaptability. **DOI:** http://dx.doi.org/10.7554/eLife.06956.002

ILLUSTRATION: www.claudiastocker.com.

As these articles highlight, many model organisms have a boom-and-bust lifestyle: *C. elegans* and *D. melanogaster*, for example, both colonize rotting food sources in the wild, reproducing rapidly when food is available. As a result, they have rapid life cycles while food is abundant, making generation times short, which is an ideal attribute for studying heritable traits and for generating experimental populations. In the wild, *A. thaliana* is highly successful at reproducing rapidly in poor habitats when environmental conditions are good. Its resulting short generation time, combined with its ability to self fertilize and its small condensed genome, make it an ideal plant for research purposes.

But selecting for these highly-favoured, rapid-cycling traits has consequences. It means that some of our favourite models tend to be ‘ecological escape artists’ that avoid rather confront the selection pressures imposed by predators, the environment and competitors—pressures that longer-lived, slower-growing species must cope with through adaptation. Unfortunately, the ability of some model organisms to escape selection pressures has severely limited the traits that can be studied in these systems, meaning that ecologists and evolutionary biologists are often not able to study the traits they are most interested in. *Arabidopsis*, for example has a wonderfully simple root system, which is ideal for imaging developmental processes, but it lacks the structural complexity that is likely to be important for plants with non-ruderal lifestyles. Moreover, *Arabidopsis* does not have any associations with mycorrhizal fungi, a symbiotic association of great importance for most land plants, and of significant agricultural importance too. And as a successful self fertilizer, its flowers are also of little interest to pollinators and, consequently, to the biologists who investigate them.

## Genomics to the rescue

With the availability of inexpensive short-read sequencing methods, literally hundreds of strains of many supermodel organisms have been sequenced and assembled, providing population biologists with the data they require to conduct ‘reverse ecology’ (Li et al., 2008). This approach uncovers genomic imprints left by past selection pressures, offering insights into trait evolution in the course of an organism's natural history. It also affords new opportunities to test directly associations between genotype and phenotype. But researchers still need to discover and collect the most informative strains to sequence, and this requires good old-fashioned field work.

The authors of the articles in this series represent a growing generation of systems biologists who are seeking out the still-feral relatives of our laboratory models. Their articles highlight that much remains to be learnt about the basic ecology of these model organisms and they provide a tantalizing view of the new frontiers that remain to be explored when we place these well-investigated organisms, and our knowledge of their biology, into a natural history context.

Surprisingly, we know very little about the real lives of many model organisms, but it is clear that some of them are not very representative of their species.

For example, *S. cerevisiae* has been found in the bark of oak trees and in primary forests in China that are remote from human activity. Perhaps it is only a coincidence that wine makers have favoured oak as a material for constructing wine barrels and casks. More interestingly, *S. cerevisiae* has also been found in the faecal human microbiome in particular populations, suggesting a much more intimate association with humans than had been previously suspected.

Knowing the natural habitats of model organisms is essential for understanding key aspects of their biology. Habitats can shape behaviours, such as foraging and shoaling, and can inform our understanding of life cycles and predator-prey relationships, as well as an organism's ability to adapt to different environmental conditions. By understanding habitat and lifestyle we can also gain insights into the processes that shape the genomes of the organisms used in research, a key feature of their biology. The sampling of wild populations of zebrafish has revealed, for example, that they contain extensive levels of genetic diversity, only a fraction of which is seen in lab strains of these fish.

## In the future everyone can be a model

Recent advances in high-throughput, genome-wide approaches and in genome-editing technologies, such as CRISPR/CAS9, allow the genomes of non-model and wild organisms to be manipulated (Chen et al., 2014; Doudna and Charpentier, 2014). As such, the particular traits that made our model organisms easy to domesticate need no longer limit the scope of scientific inquiry. The consequent blurring of the model/non-model boundary could, in the future, bring together currently disparate biological faculties, uniting them in their search to understand the important traits that are distributed throughout all biodiversity.

Our planet's biological legacy lies in the astounding array of solutions that evolutionary processes have found for all of the challenges that organisms face in all habitats on this planet. In the not-too-distant future, we will need to harvest food and other resources from most habitats on earth and to produce substantially more efficient crops to sustain our burgeoning human population (Ronald, 2014). And this will require a deeper understanding of the genetic traits that have made some organisms particularly efficient, stress-tolerant and clever at solving ecological challenges. Achieving this aim will require us to develop a unified approach to biology, one that marries the skills of ecologists and organismal biologists with those of molecular biologists. We hope that the articles in this series will help to bring these potential partners together.
